# Inappropriate prescription of cough remedies among children hospitalised with respiratory illness over the period 2002–2015 in Kenya

**DOI:** 10.1111/tmi.12831

**Published:** 2017-01-10

**Authors:** Michuki Maina, Samuel Akech, Paul Mwaniki, Susan Gachau, Morris Ogero, Thomas Julius, Phillip Ayieko, Grace Irimu, Mike English

**Affiliations:** ^1^ KEMRI‐Wellcome Trust Research ProgrammeNairobiKenya; ^2^ Department of Paediatrics and Child HealthUniversity of NairobiNairobiKenya; ^3^ Nuffield Department of MedicineUniversity of OxfordOxfordUK

**Keywords:** cough medicines, respiratory tract infection, hospitalised children, prescription practices, Kenya

## Abstract

**Objective:**

To examine trends in prescription of cough medicines over the period 2002–2015 in children aged 1 month to 12 years admitted to Kenyan hospitals with cough, difficulty breathing or diagnosed with a respiratory tract infection.

**Methods:**

We reviewed hospitalisation records of children included in four studies providing cross‐sectional prevalence estimates from government hospitals for six time periods between 2002 and 2015. Children with an atopic illness were excluded. Amongst eligible children, we determined the proportion prescribed any adjuvant medication for cough. Active ingredients in these medicines were often multiple and were classified into five categories: antihistamines, antitussives, mucolytics/expectorants, decongestants and bronchodilators. From late 2006, guidelines discouraging cough medicine use have been widely disseminated and in 2009 national directives to decrease cough medicine use were issued.

**Results:**

Across the studies, 17 963 children were eligible. Their median age and length of hospital stay were comparable. The proportion of children who received cough medicines shrank across the surveys: approximately 6% [95% CI: 5.4, 6.6] of children had a prescription in 2015 *vs*. 40% [95% CI: 35.5, 45.6] in 2002. The most common active ingredients were antihistamines and bronchodilators. The relative proportion that included antihistamines has increased over time.

**Conclusions:**

There has been an overall decline in the use of cough medicines among hospitalised children over time. This decline has been associated with educational, policy and mass media interventions.

## Abstract (fr)

**Objectif:**

Examiner les tendances de la prescription de médicaments contre la toux durant la période de 2002 à 2015 chez les enfants de 1 mois à 12 ans admis dans les hôpitaux kenyans avec de la toux, des difficultés respiratoires ou ayant reçu un diagnostic d'infection des voies respiratoires.

**Méthodes:**

Nous avons examiné les dossiers d'hospitalisation des enfants inclus dans quatre études fournissant des estimations transversales de la prévalence dans les hôpitaux publics pour 6 périodes entre 2002 et 2015. Les enfants atteints d'une maladie atopique ont été exclus. Parmi les enfants admissibles, nous avons déterminé la proportion ayant été prescrite tout médicament adjuvant contre la toux. Les principes actifs de ces médicaments étaient souvent multiples et ont été classés en cinq catégories: antihistaminiques, antitussifs, mucolytiques/expectorants, décongestionnants et bronchodilatateurs. Depuis la fin de 2006, des directives découragent l'usage de médicaments contre la toux ont été largement diffusées et en 2009, des directives nationales visant à diminuer l'utilisation des médicaments pour la toux ont été publiées.

**Résultats:**

Au cours des études, 17 963 enfants étaient admissibles. Leur âge médian et la durée de leur hospitalisation étaient comparables. La proportion d'enfants qui ont reçu des médicaments contre la toux a chuté dans les enquêtes: environ 6% [IC95%: 5,4 ‐ 6,6] des enfants avaient une ordonnance en 2015 contre 40% [IC95%: 35,5 ‐ 45.6] en 2002. Les principes actifs les plus courants étaient des antihistaminiques et des bronchodilatateurs. La proportion relative comprenant des antihistaminiques a augmenté au fil du temps.

**Conclusions:**

Il y a eu une baisse générale de l'utilisation de médicaments contre la toux chez les enfants hospitalisés au fil du temps. Ce déclin a été associé à des interventions éducatives, politiques et de média de masse.

## Abstract (es)

**Objetivo:**

Examinar las tendencias en la prescripción de medicamentos para la tos dentro del periodo entre 2002 y 2015 en niños con edades entre 1 mes y 12 años admitidos en hospitales de Kenia con tos, dificultad respiratoria o diagnosticados con una infección del tracto respiratorio.

**Métodos:**

Hemos revisado las historias clínicas de niños incluidos en cuatro estudios, obteniendo un cálculo de prevalencia croseccional de hospitales gubernamentales dentro de 6 periodos de tiempo entre el 2002 y el 2015. Se excluyeron los niños con una enfermedad atópica. Entre aquellos elegibles, determinamos la proporción prescrita de cualquier medicamento para la tos. Los ingredientes activos en estos medicamentos eran a menudo múltiples y se clasificaron en cinco categorías: antihistamínicos, antitusivos, mucolíticos/expectorantes, decongestionantes y broncodilatadores. Desde finales del 2006, las guías que desaconsejan el uso de medicamentos para la tos han sido ampliamente diseminadas y en el 2009 se lanzaron directivas nacionales para disminuir el uso de medicamentos para la tos.

**Resultados:**

A lo largo de todos los estudios 17,963 niños fueron elegible. Se compararon la mediana de edad y el tiempo de hospitalización. La proporción de niños que recibieron medicamentos para la tos disminuyó a lo largo de los estudios: aproximadamente un 6% [IC 95% 5.4, 6.6] de los niños tenían una prescripción en el 2015 vs. 40% [IC 95% 35.5, 45.6] en el 2002. Los ingredientes activos más comunes eran antihistamínicos y broncodilatadores. La proporción relativa que incluía antihistamínicos ha disminuido a lo largo del tiempo.

**Conclusiones:**

Ha habido una disminución general en el uso de medicamentos para la tos entre los niños hospitalizados a lo largo del tiempo. Esta disminución se ha asociado a intervenciones educativas, políticas y de medios de comunicación masiva.

## Introduction

Cough is a common symptom in children presenting with respiratory tract infections. It tends to cause anxiety to caregivers, discomfort to the child 1, and physicians are usually under pressure to prescribe medication for symptomatic relief contrary to treatment guidelines 2, 3, 4. Medications used include mucolytic agents (to reduce the thickness of mucus), decongestants and antitussives (to suppress cough). Cough suppression may lead to accumulation of secretions, airway obstruction and hypoxaemia 5, 6. Some cough medications contain active ingredients that have been associated with serious adverse reactions including arrhythmias, behavioural disturbances, respiratory depression and even death 5, 7, 8. Many studies, mainly in Europe and North America, have shown widespread use of cough mixtures, mainly decongestants and antitussive medications 9 and report that up to 6% of all visits to the emergency department could be secondary to adverse drug events in children under 12 years using cough medicines 10.

There have been varied efforts to improve care and guideline compliance for treatment of respiratory infections in Kenya over a number of years. These include the initial introduction and scale up of the Integrated Management of Childhood Illness (IMCI) from 1999, introduction and scale up of Emergency Triage Assessment and Treatment plus admission care (ETAT+) training targeting hospital‐based care from 2006 and efforts at disseminating policy through the media (2009) to discourage prescription of cough mixtures to children 11, 12. There have, however, been few reports on the use of cough medication in children in the sub‐Saharan region, and most have examined their use in outpatient settings 13. Three prior studies in Kenyan hospitals using standardised methods 14, 15, 16, 17 and ongoing inpatient surveillance in paediatric wards in 13 Kenyan county hospitals 18 present an opportunity to examine trends in prescription of cough mixtures over the period 2002–2015 in ill children admitted to hospital for whom risks of adverse effects may be higher. Although behaviour change at scale may take time to manifest, it is unusual to have longitudinal data from a low‐income setting. This report presents such data that may be of wider relevance given current concerns on how to reduce unnecessary antibiotic use.

## Methods

### Setting

The study uses data collected using a consistent method as part of four research studies in Kenya that provide data from six time points between 2002 and 2015 15, 16, 17, 18, 19. These studies were carried out in hospital facilities located in a district's (now county) main town, which provide first referral care. Surveys were conducted in collaboration with the Ministry of Health as part of research examining the quality of paediatric care. Facilities across Kenya were purposefully sampled to meet the needs of the particular survey (Table 1). Children in these facilities are expected to be under the care of general medical officers and clinical officers (non‐physician clinicians) under the supervision of a paediatrician who is responsible for inpatient prescribing, one focus of the surveys. In all the surveys, a similar approach to training data collectors to review medical records of children after discharge was used (comprising workshops, pilot exercises and use of standard operating procedures), similar data elements were collected, and data collection was supervised with procedures for quality assurance employed. We have previously shown that collecting these data from archived records provides the same results as collecting data while children are admitted 17. The data used in the analyses reported included demographic data, documented clinical signs and symptoms, vital signs, the admission drugs prescribed and the clinical outcome 20. In an effort to standardise the type of care being evaluated, we excluded one facility in the 2013–2015 survey as care is provided by non‐physician clinicians, there are no qualified doctors and it is a large demonstration health centre with few inpatient beds rather than being a county referral hospital. A summary of the surveys and the hospital selection process is shown in Table 1. All studies were approved by the Kenyan Ministry of Health and had ethical approval from the Kenya Medical Research Institute.

**Table 1:** Key features of the clinical studies that collected data from medical records of children discharged after admission with acute non‐surgical illnesses

Study name	District hospital study	Eight district hospital study		Sircle study	The clinical information network	
Period of data used	2002 15	2006 (Pre‐intervention) 28, 29	2008 (Post‐intervention) 28, 29	2012 16, 19	2013^a^ ^18^, ^30^	2015^a^ ^18^, ^30^
Number of Hospitals	14	8	8	22	13	13
Total cases examined in survey	641	8212	6624	1298	13 081	14 639
Case selection process	Convenience sample of recently hospitalised children	Simple Random sampling of paediatric inpatient records over six months period prior to survey		Convenience sample of hospital inpatient records from most recent discharges	Comprehensive sample of all paediatric hospital inpatient records from the most recent discharges. Surgical cases and newborns are excluded	
Intervention prior to the study	None	None	Provision of training on clinical guidelines, feedback and supportive supervision	None	(Ongoing Intervention) Three monthly feedback on quality of medical documentation and adherence to guidelines (no specific feedback on cough medicines)	
Number meeting inclusion criteria	383	2166	2199	891	6140	6157

a Included the first six months of enrolment into the study for each facility in the 2013 period and six months of 2015 (June–November).

### Study population and data analysis

We used de‐identified data from all children from 1 month to 12 years admitted with symptoms of cough or difficulty in breathing or a diagnosis of an upper or lower respiratory tract infection based on the ICD 10 criteria 21 (Table S1). The analysis excluded children with a diagnosis indicating an atopic illness, as these children may have been given antihistamines as part of their treatment (Table S2). We included children with a diagnosis of asthma so as to capture the (inappropriate) use of oral bronchodilators, which is currently not recommended due to the adverse effects. Inhaled bronchodilators are the treatment of choice for asthma in children 22. Amongst eligible children, we determined the proportion prescribed any adjuvant medication for cough. To do this, we matched the names of each child's prescribed drugs against published lists of medicines available in Kenya and formularies to determine their active ingredients. We classified active ingredients in these medicines (referred to as a group as cough medicines) into five categories: antihistamines, antitussives, mucolytics/expectorants, decongestants and bronchodilators 23 (Table S3). The data are summarised as the proportion of children admitted with a respiratory illness with at least one cough medicine calculating 95% confidence intervals (95% CI) around this estimate and presenting the result graphically. We further explored the pattern of active ingredients used over time using graphical methods. In these latter analyses, each active ingredient was counted such that a medicine containing three active compounds would contribute 1 count in each of three categories of ingredients.

### Ethical approval

The findings reported here derive from data collected in three studies that were individually approved by The KEMRI Scientific and Ethics Review committees.

## Results

The median age and length of the hospital stay of the participants were comparable across the data sets. However, children included less frequently had cough in later studies (2013 and 2015), and fever was more prevalent in some surveys (2006 and 2008). Mortality also varied across the studies from 4.7% to 10.2%. Table 2 summarises data on the populations of children included in each study.

**Table 2:** Demographic Characteristics across the survey data sets

Survey	2002 *N* = 383	2006 *N* = 2166	2008 *N* = 2199	2012 *N* = 891	2013 *N* = 6140	2015 *N* = 6157
Median age in months (IQR)	12 (5–28.25)	12 (6–27)	12 (6–24)	14 (8–27)	14 (7–22)	16 (8–30)
Presence of fever (%)	303 (79.11)	1625/1675^a^ (97)	1825/2085^a^ (87.5)	684 (76.8)	4653 (75.8)	4411 (71.6)
Presence of cough (%)	316 (82.51)	1643/1693^a^ (97)	1751/2075^a^ (84.4)	667 (74.9)	3958 (64.5)	3743 (60.8)
Average length of stay days (IQR)	3 (2–5)	3 (2–6)	3 (2–5)	3 (2–6)	3 (2–6)	3 (2–6)
Mortality rate (%)	22/383 (5.74)	162/2106 (7.7)	221/2162^a^ (10.2)	42 (4.7)	324 (5.3)	353 (5.7)

a Missing values were excluded in the computation, hence different denominators from the sample size.

### Cough medicine prescription trends

**Figure 1:**
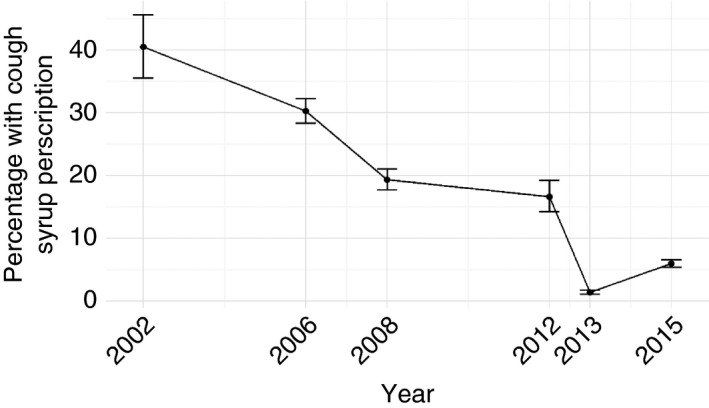
Percentage of cough medicine prescriptions across the various studies.

The proportion of children who received cough medicines diminished across the surveys with approximately 6% [95% CI: 5.4, 6.6] having a prescription in 2015 *vs*. 40% [95% CI: 35.5, 45.6] 13 years earlier. There was a marked decrease between 2012 and 2013/14; however, we note a small increase in their use between the 2013 and 2015 surveys as shown in Figure 1.

### Cough medicine contents

**Figure 2:**
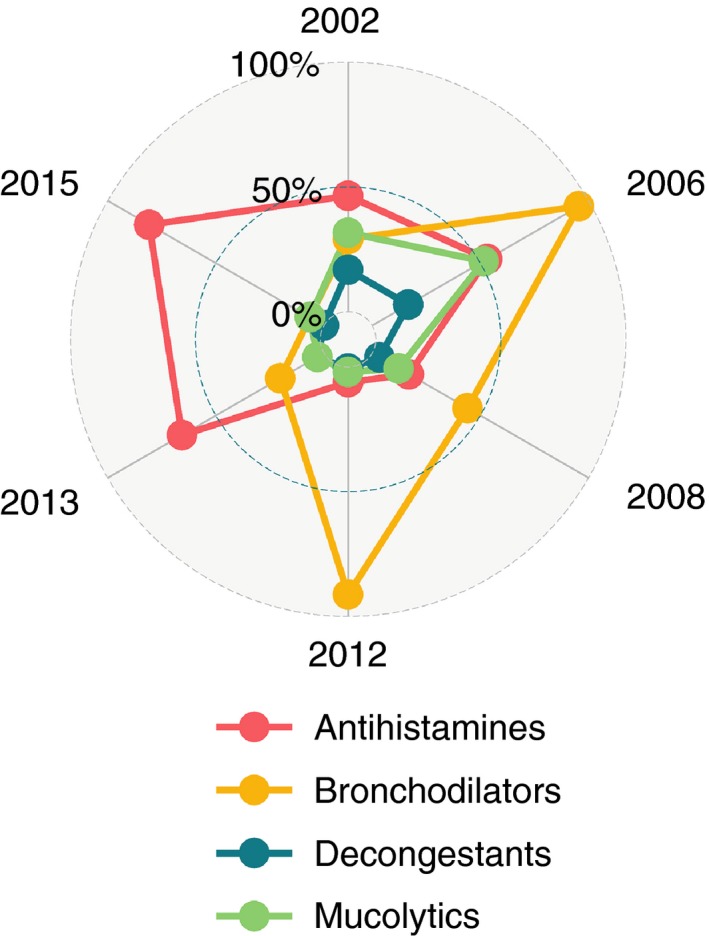
Radar plot describing the proportion of all active ingredients in cough medicines that were either antihistamines, bronchodilators, decongestants or mucolytics for a given year.

Across the surveys, the most common active ingredients in prescribed cough medicines were antihistamines and bronchodilators. Between 2006 and 2012, cough medicines containing oral bronchodilators constituted the bulk of prescriptions. Amongst children prescribed a cough medicine, the proportion that include antihistamines as active ingredients increased over time, constituting over half of the prescriptions in the 2013 and 2015 surveys. Prescription of decongestant‐containing medicines remained relatively infrequent across the studies (Figure 2). The prescriptions containing antitussives were very few and are not included in the figure.

## Discussion

There has been a general decline in the prescription rates for cough medicines across the fourteen years from 40% in 2002 to 6% in 2015 for children admitted to general district (now county) hospitals with cough, difficulty breathing or diagnosed with an upper respiratory infection. This is despite the low cost of cough medicines and continuing aggressive marketing by manufacturers in Kenya, which has been identified as one of the reasons why cough medicines are prescribed. It has also been suggested that health workers have a poor understanding of the ingredients in cough medicines 13. This suggests that marketing or some perceived benefit of active ingredients where they are known might influence changes in the pattern of active ingredients over time. Accompanying an overall decline in cough medicine prescribing was a shift across the surveys first towards bronchodilator and then towards antihistamine‐containing cough medicines. This may be due to more effective marketing of newer generation antihistamine medications said to have better safety profiles 24. In earlier surveys, children prescribed a cough medicine containing bronchodilators may often have been prescribed inhaled beta‐agonists as specific treatment too (unpublished observation). The later decline in use of cough medicines containing bronchodilators may, we speculate, be linked to efforts to improve awareness of the appropriate use of inhaled bronchodilators for asthma and that oral bronchodilators are of limited effectiveness.

Across the time period spanning the surveys and the decline in cough medicine use, there have been a number of initiatives that may have influenced prescribing habits (Table 3). In addition to these, there has been an improvement in clinical staffing numbers and the cadre of clinical staff in charge of the paediatric wards has slowly changed. In 2002, with few paediatricians in the country, most inpatient paediatric care was led by clinical officers (non‐physician clinicians) supervised by general medical officers. However, by the 2013–2015 survey, inpatient paediatric care in county hospitals, including the 13 in this study, was often led by a consultant paediatrician assisted by several intern medical officers and sometimes a general medical officer 25. Many of these medical officers and paediatricians were trained in use of and have access to national guidelines 26, with the training typically emphasising that cough medicines are contraindicated. We speculate therefore that some of the decline in use of cough medicines for the inpatient population is attributable to greater presence in hospitals of junior physicians and consultant paediatricians educated not to use cough medicines. Such staff would also have been target recipients of updated pneumonia guideline dissemination after the 2013 revisions when a notable decrease in cough medicine use occurred. (Figure 1) Interestingly, a review of the prescriptions from the excluded health facility, where all patient care is provided by non‐physician clinicians, revealed a larger number of cough medicine prescriptions amongst those admitted for care for pneumonia, averaging about 14% for the two periods (2013 and 2015).

**Table 3:** Major Interventions that may have contributed to a decline in cough medicine prescriptions

Year	Intervention	Description of intervention
1995 4, 31	Integrated management of childhood illness (IMCI)	Improving the case management of common illnesses including respiratory illnesses. Gave clear guidelines on how to effectively handle children with cough highlighting potential harm in cough mixtures.
2006 11, 26, 32	Emergency Triage Assessment and Treatment pus admission care (ETAT+)	Guidelines to assist in classification and treatment of children presenting with cough with wide‐scale national dissemination from 2008 onwards. In particular, 10 000 copies of guidelines were disseminated in 2010, and following a national pneumonia treatment policy review in 2013, 12 000 copies of updated guidelines were disseminated in 2013 and presented to over 200 paediatricians at the national paediatric conference. The guidelines and training clearly discourage the use of cough mixtures and use of oral bronchodilator medications
2009 12	Ministry of Health	Through the pharmacy and poisons board issued a policy directive on the use of cough remedies. Disseminated through print media
2009 33	Private Hospitals ban use of cough mixtures i∝n children	Largest Kenyan private children's hospital and a university hospital remove cough syrups from their hospital formularies and pharmacies, an event covered by national print media

This study had a number of limitations. Purposive sampling of hospitals was employed for each survey, and there was no pre‐specified aim to track use of unnecessary medicines across time. Each study also provided different samples sizes for analysis, and the data were collected by different teams in the individual studies even though similar tools were used. Based on the data collected, we are unable to determine the doses of the medicines prescribed, the frequency of administration or whether any adverse events occurred as a result of the prescriptions. Furthermore, it is possible that some prescriptions for cough medicines were not recorded in hospital records but given directly to parents. In the 2013–2015 survey, we noted an increase in the prescription of cough medicines between the two six‐month periods sampled. A reduction in levels of missing data on other parameters of interest within the clinical information network has been noted over this period 27. An improvement in the capture of data on cough medicines may therefore be contributing to their apparent increase in use.

Despite these limitations, we feel that the decline in the prescription of cough medicines observed across surveys in hospitalised children in Kenya is likely to reflect a true change in practice over a period of 14 years. We suggest that reducing exposure of already sick hospitalised children to their potential adverse events is likely therefore to have helped improve patient safety and reduced healthcare costs. As most cough remedies may be provided to ambulant children as over the counter or outpatient prescriptions, examination of cough mixture use among these groups of children would also be useful to provide a more complete picture of the situation in Kenya and other countries.

## Conclusion

There has been an overall decline in the use of cough medicines among hospitalised children across Kenyan hospitals over time. This decline has been associated with educational, policy and mass media interventions and an expansion of the physician and specialist paediatric workforce. We believe the findings help demonstrate the value of long‐term surveillance of prescribing habits that may be of particular value in an era when there is increasing concern around rational treatment use and stewardship.

## Supporting information


**Table S1.** ICD 10 classification of upper and lower respiratory tract conditions presenting with cough.
**Table S2.** ICD 10 classification of respiratory allergic conditions.
**Table S3**. Classification of common ingredients in cough mixtures.
