# Supplementary material for: Inappropriate prescription of cough remedies among children hospitalised with respiratory illness over the period 2002–2015 in Kenya
Source: Trop Med Int Health. 2017 Jan 10;22(3):363–9. doi: 10.1111/tmi.12831 (PMC5347920; doi:10.1111/tmi.12831)
Supplement: Supplementary file 1 — Table S1. ICD 10 classification of upper and lower respiratory tract conditions presenting with cough. Table S2. ICD 10 classification of respiratory allergic conditions. Table S3. Classification of common ingredients in cough mixtures. [file TMI-22-363-s001.docx]

**Supplementary Table 1.** ICD 10 classification of upper and lower respiratory tract conditions presenting with cough

| **ICD 10 classification** | **Diagnosis** |
| --- | --- |
| A16.2 | Tuberculosis of lung, without mention of bacteriological or histological confirmation |
| A37 | Whooping cough |
| J00 | Acute nasopharyngitis [common cold] |
| J01s | Acute sinusitis |
| J02 | Pharyngitis |
| J04.2 | Acute laryngotracheitis |
| J05 | Acute obstructive laryngitis [croup] and epiglottitis |
| J05 | Acute obstructive laryngitis [croup] and epiglottitis |
| J05.0 | Acute obstructive laryngitis [croup] |
| J06.0 | Acute laryngopharyngitis |
| J06.9 | Acute upper respiratory infection, unspecified |
| J18.0 | Broncho pneumonia |
| J18.9 | Pneumonia(No classification ) |
| J18.9A | Pneumonia(Non Severe) |
| J18.9B | Pneumonia(severe) |
| J18.9C | Pneumonia(Very severe) |
| J20.9 | Acute bronchitis, unspecified |
| J21 | Bronchiolitis |
| J31.1 | Chronic nasopharyngitis |
| J35.1 | Hypertrophy of tonsils |
| J39.9 | Upper Respiratory Tract Infection |
| J45.9B | Asthma-Severe |
| J46 | Asthma-very severe |
| J68 | Respiratory conditions due to inhalation of chemicals, gases, fumes and vapours |
| J90 | Pleural effusion, NEC |
| R06.1 | Stridor |
| R06.2 | Bronchospasm |

**Supplementary Table 2.** ICD 10 classification of respiratory allergic conditions

| J30.4 | Allergic rhinitis, unspecified" |
| --- | --- |
| T78.4 | Allergy, unspecified. |
| Z88.0 | Personal history of allergy to penicillin |
| L23 | Allergic contact dermatitis" |
| T78.4 | Allergy, unspecified |
| Z88.7 | Personal history of allergy to serum and vaccine |
| J31.0 | Chronic Rhinitis |
| J30.9 | Allergic Rhinitis, Cause Unspecified Sinus |

**Supplementary Table 3**. Classification of common ingredients in cough mixtures

| **Drug** | **Active ingredients** |  |
| --- | --- | --- |
| Antihistamines | Brompheniramine Chlorpheniramine  Dexchlorpheniramnie Diphenhydramine  Prometahzine | Doxylamine  Pheniramine  Loratidine  Cetirizine  Tripolidine |
| Antitussive | Codeine Dextromethorphan Dihydrocodeine | Pentoxyverine Pholcodine |
| Mucolytics/expectorants | Ambroxol  Ipecacuanha  senega and ammonia | Bromhexine  Guaiphenesin |
| Decongestants | Phenylephrine  Pseudoephedrine  oxymetazoline | Ephedrine  xylometazoline |
| Antipyretics in the cough medication | Paracetamol |  |
| Bronchidilators in the cough medication | Salbutamol  Terbutaline |  |

Source: Australian Government Department of Health. Cough and cold medicines for children Australia2015 [updated August 2015 cited 2015 September 1]. Available from: https://www.tga.gov.au/node/105
